# JAK抑制剂在噬血细胞性淋巴组织细胞增生症中的作用机制及临床进展

**DOI:** 10.3760/cma.j.cn121090-20251209-00578

**Published:** 2026-06

**Authors:** 孟轩 王, 云泽 赵, 志刚 李, 天有 王, 蕊 张

**Affiliations:** 1 首都医科大学附属北京儿童医院血液病科，北京 100045 Department of Hematology, Beijing Children's Hospital, Capital Medical University, Beijing 100045, China; 2 首都医科大学附属北京儿童医院血液疾病研究室，北京 100045 Hematologic Diseases Research Laboratory, Beijing Children's Hospital, Capital Medical University, Beijing 100045, China

## Abstract

噬血细胞性淋巴组织细胞增生症（HLH）是一类由淋巴细胞和巨噬细胞异常活化引发的高致死性过度炎症反应综合征。其病因具有高度异质性，可分为原发性和继发性HLH。以芦可替尼为代表的Janus激酶（JAK）抑制剂可阻断JAK-STAT信号通路，抑制γ干扰素等多种细胞因子的分泌，在多种病因背景的HLH中均显示出独特的治疗价值。本文就JAK抑制剂在HLH中的作用机制及临床研究进展进行综述。

## Abstract (en)

Hemophagocytic lymphohistiocytosis（HLH）is a life-threatening hyperinflammatory syndrome characterized by uncontrolled activation of lymphocytes and macrophages. Owing to its marked etiological heterogeneity, HLH is broadly classified into primary and secondary forms. Janus kinase（JAK）inhibitors, represented by ruxolitinib, exert therapeutic effects by blocking the JAK-STAT signaling pathway and suppressing the secretion of various cytokines, including interferon-γ. Accumulating evidence suggests that JAK inhibitors demonstrate unique therapeutic potential across diverse etiological backgrounds of HLH. This review summarizes the mechanisms and clinical research progress of JAK inhibitors in the treatment of HLH.

噬血细胞性淋巴组织细胞增生症（HLH）是由淋巴细胞过度活化及细胞因子风暴如γ干扰素（IFN-γ）、白细胞介素（IL）-6等引发的致死性炎症反应综合征[1]。若缺乏及时干预，患者常迅速进展至多器官衰竭[2]。HLH-1994与HLH-2004方案长期作为HLH一线治疗方案，主要针对原发性HLH（pHLH），3年总生存（OS）率可达82％[3]。但对于病因异质性强的继发性HLH（sHLH），其疗效和安全性仍存在局限。Janus激酶-信号转导及转录激活因子（JAK-STAT）信号通路在HLH发病中起着关键作用，因此，能广泛阻断多种炎症因子的JAK抑制剂（JAKi）成为精准治疗的新选择。本文旨在综述JAKi治疗HLH的作用机制及最新临床研究进展。

一、JAKi治疗HLH的机制

JAK-STAT信号通路在HLH的免疫失衡放大与维持中扮演关键角色。JAK家族成员（JAK1/2/3、TYK2）通过介导IFN-γ、IL-2、IL-6等促炎因子的信号转导，驱动细胞因子风暴的形成。JAKi通过竞争性结合三磷酸腺苷（ATP）位点，阻断STAT蛋白磷酸化，从而切断炎症信号级联反应[4]。基于动物模型及转录组学研究，其机制主要包括以下4点（图1）。

**图1:**
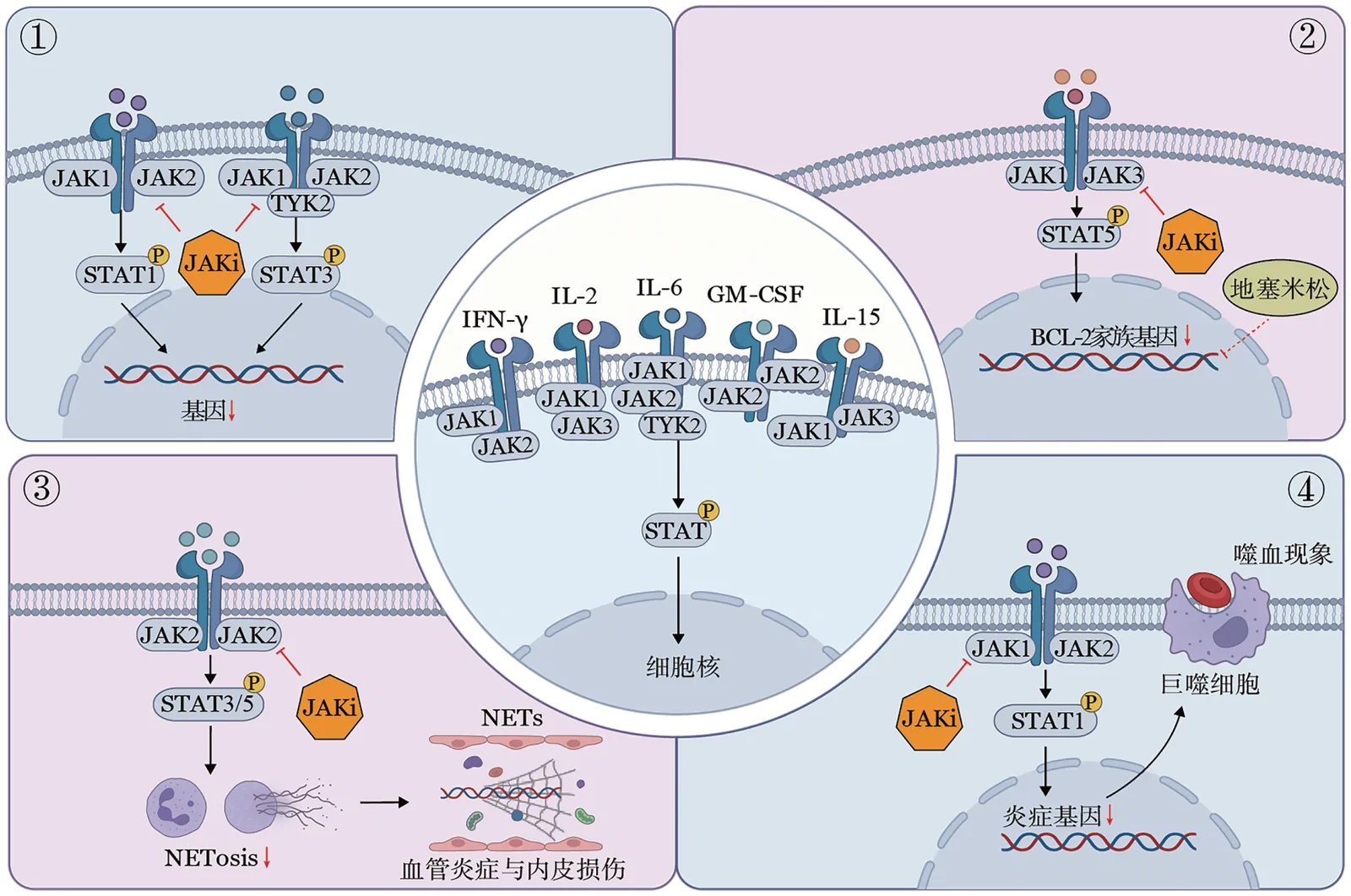
JAK-STAT信号通路在噬血细胞性淋巴组织细胞增生症中的作用机制 **注** JAK：Janus激酶；STAT：信号转导及转录激活因子；JAKi：JAK抑制剂；TYK2：酪氨酸激酶2；IFN-γ：γ干扰素；IL：白细胞介素；GM-CSF：粒细胞-巨噬细胞集落刺激因子；TNF-α：肿瘤坏死因子α；BCL-2：B细胞淋巴瘤-2；NETs：中性粒细胞胞外诱捕网；NETosis：NETs形成过程。①阻断IFN-γ依赖及非依赖性炎症环路：JAKi通过抑制JAK1/JAK2活性，下调STAT1（IFN-γ通路）及STAT3（IL-6等非依赖性通路）磷酸化，减少下游促炎基因转录；②逆转T细胞凋亡耐受并恢复激素敏感性：JAKi抑制IL-2/IL-15-JAK1/JAK3-STAT5信号轴，降低BCL-2家族基因的表达，与地塞米松协同恢复细胞凋亡敏感性；③抑制中性粒细胞活化与NETs形成：JAKi通过抑制GM-CSF介导的JAK2-STAT5信号通路，减少中性粒细胞NETosis，减轻血管炎症及内皮损伤；④抑制巨噬细胞过度活化与T细胞-巨噬细胞环路：JAKi阻断IFN-γ介导的JAK1/JAK2-STAT1信号，下调炎症基因转录，进而减少巨噬细胞浸润及噬血现象。图例说明：实线箭头表示激活；平头线表示抑制；虚线表示间接过程；↓表示JAKi作用后表达或活性下降；橙色五边形表示JAKi；黄色椭圆表示地塞米松

（一）阻断IFN-γ依赖及非依赖性炎症环路

Das等[5]在淋巴细胞性脉络丛脑膜炎病毒感染的穿孔素全身敲除（Prf1^−^/^−^）pHLH小鼠模型中证实，JAK1/2抑制剂芦可替尼（RUX）可通过抑制STAT1磷酸化，从而阻断IFN-γ信号转导。Albeituni等[6]进一步阐明，即使完全中和IFN-γ后，炎症反应仍可持续；而RUX通过同时阻断IL-6、粒细胞-巨噬细胞集落刺激因子（GM-CSF）等IFN-γ非依赖性细胞因子信号，能显著降低残留炎症负荷。

（二）逆转T细胞凋亡耐受并恢复激素敏感性

Meyer等[7]报道，HLH患者体内升高的STAT5相关细胞因子（如IL-2、IL-7、IL-15）通过JAK/STAT通路，上调CD8^+^T细胞中B细胞淋巴瘤-2（BCL-2）家族抗凋亡蛋白（如BCL-2、BCL-xL等）的表达，导致凋亡抵抗。RUX可抑制该信号轴，降低抗凋亡蛋白表达水平，恢复细胞凋亡敏感性，并在Prf1^−^/^−^模型中与地塞米松呈协同作用。

（三）抑制中性粒细胞活化与中性粒细胞胞外诱捕网（NETs）形成

JAKi亦参与固有免疫调控。Ma等[8]结合Padi4^−^/^−^小鼠模型及体外实验表明，JAK-STAT通路参与驱动中性粒细胞过度活化及NETs形成（NETosis）。RUX通过抑制STAT3/5磷酸化，显著减少NETosis。鉴于NETosis与血管炎症、内皮损伤及微循环障碍密切相关，其受抑或有助于减轻HLH相关组织损害[9]。

（四）抑制巨噬细胞过度活化与T细胞-巨噬细胞环路

巨噬细胞的异常活化及噬血现象是HLH的典型病理特征，其持续激活依赖于IFN-γ及GM-CSF等信号。JAKi通过阻断STAT1/3介导的炎症转录程序抑制巨噬细胞活化，减少IL-6、肿瘤坏死因子（TNF）-α等促炎因子释放。Albeituni等[6]及Keenan等[10]的动物研究显示，抑制JAK1/2激酶活性可减少组织内巨噬细胞浸润并改善脏器炎症损伤，提示其能够从效应细胞层面切断T细胞与巨噬细胞之间的恶性循环，抑制细胞因子风暴。

二、JAKi在HLH中的临床研究进展

随着研究的深入，JAKi在HLH中的应用正由挽救治疗逐渐拓展至更广泛的临床场景。以RUX为代表的研究初步显示出其在多种病因背景下的HLH中可快速控制炎症并减少化疗暴露的潜力（表1）。

**表1:** JAK抑制剂在不同病因噬血细胞性淋巴组织细胞增生症（HLH）中的临床研究总结

JAK抑制剂	作用靶点	研究来源	适用HLH类型	研究类型	样本量（例）	疗效	安全性
芦可替尼	JAK1/2	Zhang等[11]	EBV-HLH	Ⅱ期临床试验	24（总共52例）	28 d ORR为87.5％；RUX单药CR率为58.3％；1年OS率为87.3％	RUX单药组1例3级胰腺损伤；联合治疗组3/4级AE以骨髓抑制、严重感染为主（为全队列数据）
		Song等[12]	R/R HLH	回顾性研究	8	50％患者RUX剂量递增后达CR；2个月OS率为75％	无≥3级AE
		Ge等[13]	pHLH	队列研究	21	8周ORR为100％；CR率为90.5％；1年OS率为90.5％；38.1％未接受过化疗；81％成功桥接HSCT；移植后生存率为88.2％	≥3级AE以骨髓抑制为主，少数感染、胰腺损伤
		Zhang等[14]	EBV-HLH	临床试验	8（总共12例）	28 d ORR为100％，CR率为75％，PR率为25％	无≥3级AE
		Wang等[15]	EBV-HLH	队列研究	53	8周ORR为84.9％，1年OS率为92.2％；56.6％患者免依托泊苷	骨髓抑制及继发感染发生率显著低于化疗组；无药物相关死亡
		Fang等[16]	AD/AID-HLH	队列研究	26	8周ORR为96.2％、CR率为92.3％；2年OS率为96.2％	常见≥3级AE为骨髓抑制和病毒感染，经治疗均恢复；无治疗相关死亡
		Zhou等[17]	mHLH	Ⅱ期临床试验	16（总共28例）	ORR为75％；2个月OS率为75％	总体耐受性良好，无因不良反应减药或停药
		Gu等[18]	IEC-HS	病例报告	3	控制严重CRS，不影响CAR-T细胞治疗疗效	未提及
托法替布	JAK1/3	Liu等[19]	pHLH	病例报告	1	RUX治疗无效后改用托法替布，最终达CR	未提及
		Xie等[20]	AD/AID-HLH	病例对照研究	17（总共36例）	2周ORR为100％，传统治疗组ORR为78.9％；6周CR率为76.5％；12周激素停用率为100％，传统治疗组为57.9％	无严重AE
巴瑞替尼	JAK1/2	Yi等[21]	AD/AID-HLH	病例报告	1	激素减量时疾病复发，换用巴瑞替尼后缓解，1.5年随访无复发	无严重AE
伊他替尼	JAK1	Frigault等[22]	IEC-HS	Ⅱ期临床试验	63	≥2级CRS发生率为22.2％	≥3级AE以血液学不良反应为主，未观察到非预期的严重药物不良反应
				随机对照研究	23（总共46例）	≥2级CRS发生率为17.4％；6个月ORR为39.1％	AE总体可控，无治疗相关死亡

**注** EBV-HLH：EB病毒相关性HLH；R/R HLH：难治/复发性HLH；pHLH：原发性HLH；AD/AID-HLH：自身免疫/自身炎症性疾病相关HLH；mHLH：恶性肿瘤相关HLH；IEC-HS：免疫效应细胞相关HLH样综合征；ORR：客观缓解率；CR：完全缓解；OS：总生存；HSCT：造血干细胞移植；PR：部分缓解；CRS：细胞因子释放综合征；RUX：芦可替尼；CAR-T细胞：嵌合抗原受体T细胞；AE：不良事件

（一）难治/复发性HLH（R/R HLH）

R/R HLH是指对传统常规一线治疗方案（如HLH-1994或HLH-2004方案）反应不佳、未能达到病情缓解，或在缓解后疾病复发的高危疾病状态。RUX最早用于对传统治疗无效的R/R HLH患者。病例报告及小样本研究显示，部分R/R HLH患者接受RUX治疗后可于24 h内退热，在1～2周内铁蛋白、可溶性CD25等炎症指标显著改善，并成功桥接造血干细胞移植（HSCT）[23]–[24]。尽管部分研究中客观缓解率（ORR）可达70％[25]–[26]，但持续缓解率较低，长期疾病控制效果有限。

在R/R HLH中，JAKi主要作为挽救治疗（二线及以上）应用。对于常规剂量RUX反应不佳的R/R HLH患者，Song等[12]研究提示，采用剂量递增方案（最大剂量可达30 mg每日3次；部分联合CYP450抑制剂以提升药物浓度）可使50％患者达到完全缓解（CR），且耐受性良好。此外，JAK1/3抑制剂托法替布在难治性巨噬细胞活化综合征（MAS）中亦展现出挽救治疗潜力[20],[27]。

总之，以RUX为代表的JAKi是一线治疗方案失败后的R/R HLH患者重要的挽救治疗选择。但需强调，R/R HLH的难治性往往源于潜在病因未能得到有效控制，例如慢性活动性EB病毒（EBV）感染（CAEBV）、隐匿性恶性肿瘤或严重的原发性免疫缺陷等。临床应将JAKi定位为稳定病情和为对因治疗创造窗口的过渡性策略，而非长期维持手段。

（二）pHLH

pHLH是由遗传性免疫缺陷引起的HLH类型，主要与细胞毒性T细胞和自然杀伤细胞功能障碍相关，多由PRF1、UNC13D、STX11、STXBP2、RAB27A、LYST、SH2D1A及BIRC4等基因突变所致，多发生于婴幼儿及青少年，具有家族聚集性或早发特点[1],[28]。目前HSCT是唯一根治手段，而移植前的疾病控制对预后至关重要。传统高强度化疗虽能短期控制病情，但显著增加了移植前死亡及继发性肿瘤等发生风险[29]–[30]。

在pHLH中，JAKi主要定位为移植前的桥接治疗。2024年，Ge等[13]的分层方案实现了90.5％的1年OS率，使近40％的患者完全避免了依托泊苷（VP16）暴露，在控制疾病的同时大幅降低了化疗相关的移植前死亡与器官毒性发生风险，从而提高了成功桥接HSCT的比例，显示出良好的安全性与有效性。

基于RUX的分层治疗方案为pHLH移植前管理提供了从“强力压制”到“精准调控”的转变，具有减少不良反应、改善耐受性及提高移植成功率的潜力。但需注意，RUX穿透血脑屏障能力有限。针对pHLH中较为常见的中枢神经系统受累时，临床上常需与其他易透过血脑屏障的药物联合应用，以实现疾病控制。

此外，其他JAKi尚处探索阶段：已有个案报道携带PRF1与UNC13D复合杂合突变的老年pHLH患者在对RUX反应欠佳后改用托法替布取得了良好缓解，但由于证据等级较低，其临床意义仍需更多系统研究验证[19]。

（三）sHLH

sHLH是由多种免疫刺激因素所诱发的HLH，其发病率远高于pHLH。sHLH的病因异质性高，主要包括感染（东亚地区以EBV最为常见）、恶性肿瘤（尤其是淋巴瘤）、自身免疫/炎症性疾病（如全身型幼年特发性关节炎等）以及医源性因素［如嵌合抗原受体T（CAR-T）细胞治疗］等[1]。sHLH的治疗重点在于平衡炎症控制与原发病处理，因此亟需个体化的治疗。

1. EBV相关性HLH（EBV-HLH）：EBV-HLH是我国儿童HLH中最常见的一种类型，约占60％[31]。与一般感染相关HLH不同，其常伴持续性病毒复制及免疫细胞克隆性异常，起病急、炎症反应剧烈、复发风险高。传统HLH-94方案虽为标准治疗，但化疗相关骨髓抑制与继发感染风险显著，因此在该人群中应用受限。

在EBV-HLH中，JAKi正由二线治疗逐渐向一线治疗拓展，强调个体化分层治疗。Zhang等[14]的早期单中心试验初步显示该亚组对RUX高度敏感；其后扩大样本的分层治疗研究进一步证实部分EBV-HLH患儿仅接受RUX单药治疗即可达持续完全缓解，无需化疗[11]。Wang等[15]的对照研究进一步表明以RUX为基础的方案在生存获益上与改良HLH-94方案化疗相当，且治疗相关不良反应明显较低，尤其在骨髓抑制和继发感染风险方面。此外，56.6％的患儿未使用VP16，仅通过RUX单药或联合激素实现持续缓解，其中93.3％为原发性EBV感染，提示这一病因亚组更可能从低强度治疗中获益。

目前在EBV-HLH的治疗中JAKi的临床证据主要集中于RUX，其他JAKi的应用尚未见报道。未来需进一步探索JAKi与抗病毒治疗的联合策略，以及不同JAKi之间的疗效差异。

2. 自身免疫/自身炎症性疾病相关HLH（AD/AID-HLH）：AD/AID-HLH是一种由自身免疫病（AD）或自身炎症性疾病（AID）触发的致命性细胞因子风暴综合征，临床上亦称为MAS[32]。其病理特征为高水平炎症因子（尤其是IFN-γ、IL-6及IL-18等）驱动的单核-巨噬细胞系统失控性活化及广泛的噬血现象[33]。

在AD/AID-HLH中，JAKi主要用于激素抵抗或激素依赖患者的二线治疗。据Fang等[16]的研究显示，基于RUX的分层治疗策略在儿童AD/AID-HLH中具有显著疗效，8周ORR与CR率分别达96.2％和92.3％，2年OS率达96.2％。其中，RUX单药治疗可使46.1％的患者达到CR，中位起效时间仅2 d，体现了其迅速控制炎症的优势。安全性方面，该研究显示不良事件总体可控，最常见为骨髓抑制和感染，未观察到治疗相关的死亡事件。RUX单药治疗起效迅速，可在数日内控制炎症，对于进展凶险的细胞因子风暴尤为关键；同时具备辅助激素减量的作用，有助于避免长期使用大剂量激素所引起的不良反应。

除RUX外，其他JAKi也逐渐展现出临床潜力。JAK1/3抑制剂托法替布在一项单中心回顾性研究中较传统用药展现出更快的炎症控制与更强的激素减停能力，且未观察到血栓或严重感染事件[20]。另有个案报道提示JAK1/2抑制剂巴瑞替尼可有效控制结节性脂膜炎继发MAS患者的炎症反应，并在糖皮质激素减量期间维持病情稳定，患者随访1.5年未见严重不良反应[21]。

3. 恶性肿瘤相关HLH（mHLH）：mHLH是指由恶性肿瘤本身或其治疗过程诱发的严重高炎症反应，是sHLH预后最差的亚型之一。一项前瞻性研究显示mHLH患者1年OS率不足40％[34]。mHLH的治疗核心在于快速控制原发肿瘤。然而，失控的细胞因子风暴常导致患者一般状况急剧恶化，使其无法耐受抗肿瘤治疗[35]。

在此背景下，JAKi的作用主要定位于快速控制高炎症状态，为后续抗肿瘤治疗创造关键窗口期。Zhou等[17]针对病因未明的新诊断成人HLH［包括16例淋巴瘤相关HLH（LAHS）］的前瞻性Ⅱ期研究证实，Ru-D（RUX联合地塞米松）方案作为一线诱导治疗可有效改善mHLH患者短期预后。

需强调的是，JAKi本身不具有直接抗肿瘤活性，需与及时、有效的原发病治疗相结合才能体现其临床价值[36]。对于mHLH，探索JAKi与化疗或免疫检查点抑制剂等方案的联合应用，或许是未来的重要研究方向。

4. CAR-T细胞治疗相关HLH：随着CAR-T细胞治疗的广泛应用，部分患者可出现严重的系统性高炎症反应。美国移植与细胞治疗学会（ASTCT）于2023年发布专家共识，将该并发症统一命名为免疫效应细胞相关HLH样综合征（IEC-HS），并明确其为独立于典型细胞因子释放综合征（CRS）的临床实体[37]。目前临床多采用糖皮质激素联合IL-1受体拮抗剂阿那白滞素作为基础治疗，但部分患者疗效有限，仍需探索新的干预手段。

近年来，JAKi在CAR-T细胞治疗相关严重炎症反应中的应用逐渐受到关注，在IEC-HS中的应用包括救治与预防两种策略。作为救治用药，Gu等[18]报道，RUX可在不影响CAR-T细胞疗效的前提下有效控制严重CRS。Huarte等[38]的临床前研究亦证实，JAK1抑制剂伊他替尼能够显著降低多种炎症因子释放，同时保留CAR-T细胞增殖功能。作为预防用药，Frigault等[22]开展的随机、双盲、安慰剂对照的Ⅱ期临床试验（NCT04071366）证实，在CAR-T细胞围输注期，预防性给予伊他替尼，可将≥2级CRS的发生率从安慰剂组的56.5％显著降低至17.4％（*P*＝0.003），且不影响6个月ORR，提示其可有效预防IEC-HS且未削弱CAR-T细胞的长期抗肿瘤疗效。鉴于CRS与IEC-HS在细胞因子谱及炎症放大机制方面存在部分重叠，上述研究结果亦为JAKi在IEC-HS中的潜在应用提供了间接支持。

三、总结与展望

JAKi通过抑制JAK-STAT信号通路，可从多个层面阻断炎症级联反应，迅速控制HLH的高炎症状态。特别是以RUX为代表的JAK1/2抑制剂在HLH治疗中，对于EBV-HLH、AD/AID-HLH等sHLH患者具有较好的治疗效果和安全性。现有临床数据可初步支持基于RUX的分层治疗方案在提高HLH患者缓解率、延长生存期以及减少治疗相关不良反应方面的优势。然而，整体证据仍以小样本、回顾性研究为主。此外，不同JAKi之间的差异、最佳剂量与疗程、不同HLH亚型中的适用性，以及对长期预后的影响仍需更高质量的前瞻性研究进一步验证。

后续研究应着重于寻找能够预测JAKi疗效和预后的新型生物标志物，以实现更精确的个体化治疗。同时，还应积极探索JAKi与IFN-γ抑制剂（如依马利尤单抗）、IL-1抑制剂（如阿那白滞素）或免疫检查点抑制剂等新型靶向药物的联合应用，旨在控制炎症风暴的同时进行对因治疗，改善R/R HLH的长期预后。
